# Real-world use of hydrocortisone and dexamethasone in the prevention and treatment of bronchopulmonary dysplasia in preterm infants <32 weeks: a nine-year cohort study

**DOI:** 10.3389/fped.2026.1840133

**Published:** 2026-05-12

**Authors:** Kamal Ali, Talal Aljarbou, Saleh Algarni, Maryam Alkaabi, Abdulrahman Mandurah, Haitham Alasmari, Saad Alshareedah, Ashwag Alsubaie, Mashael Almutairi, Rana Almuqati, Ibrahim Alanazi, Shaimaa Halabi, Omar Almutairi, Abdulaziz Homedi, Saif Alsaif

**Affiliations:** 1 Neonatal Intensive Care Department, King Abdulaziz Medical City – Riyadh, Ministry of National Guard Health Affairs, Riyadh, Saudi Arabia; 2 College of Medicine, King Saud Bin Abdulaziz University for Health Sciences, Riyadh, Saudi Arabia; 3 King Abdullah International Medical Research Center, Riyadh, Saudi Arabia

**Keywords:** bronchopulmonar dysplasia, outcome, postnatal, preterm, steroids

## Abstract

**Objectives:**

To describe real-world patterns of postnatal corticosteroid use and evaluate associations with respiratory outcomes in preterm infants <32 weeks’ gestation.

**Methods:**

This retrospective cohort study included infants <32 weeks admitted to a tertiary neonatal intensive care unit at King Abdulaziz Medical City, Riyadh, Saudi Arabia, between January 2017 and December 2025. Postnatal steroid exposure was categorized as hydrocortisone, dexamethasone, both, or none. Primary outcomes were bronchopulmonary dysplasia (BPD) severity at 36 weeks’ postmenstrual age, death before 36 weeks, and a composite of BPD or death. Multivariable regression models adjusted for baseline clinical factors, and propensity score weighting was used to address confounding by indication.

**Results:**

Among 1,228 infants, 326 (26.5%) received postnatal steroids (hydrocortisone 9.5%, dexamethasone 15.2%, both 1.8%). Steroid use increased with decreasing gestational age (54.4% at <26 weeks vs. 7.0% at 30–31 weeks, *p* < 0.001) and was more frequent in infants requiring invasive respiratory support at admission. In adjusted analyses, steroid exposure was associated with higher BPD severity [adjusted odds ratio (aOR) 2.71, 95% CI 1.92–3.81], increased home oxygen (aOR 6.36, 95% CI 1.47–27.64), and longer duration of invasive ventilation [incidence rate ratio (IRR) 2.65, 95% CI 2.25–3.11]. Steroid exposure was associated with lower odds of death before 36 weeks (aOR 0.25, 95% CI 0.15–0.41), although this likely reflects treatment selection and survivor bias. Formulation-specific analyses showed higher BPD severity with combined exposure (aOR 5.67, 95% CI 2.40–13.39). Associations with the composite outcome were consistent across models and confirmed in propensity-weighted analyses (aOR 1.90, 95% CI 1.53–2.34).

**Conclusions:**

Postnatal corticosteroids were primarily used in infants with severe respiratory disease and were associated with higher observed respiratory morbidity rates in this higher-risk population. These findings reflect real-world corticosteroid use in higher-risk infants and should be interpreted as associations within a selected population rather than causal treatment effects.

## Introduction

Bronchopulmonary dysplasia (BPD) is a major complication of extreme prematurity and is associated with prolonged respiratory support, extended hospitalisation, and increased healthcare utilization during infancy and childhood. Despite advances in perinatal and neonatal care, its incidence among very preterm infants remains high across neonatal networks worldwide (1–5). The condition reflects disrupted lung development driven by the combined effects of mechanical ventilation, oxygen exposure, and inflammation, resulting in impaired alveolar and vascular growth (1, 2, 6).

Systemic corticosteroids have long been used in neonatal practice to reduce pulmonary inflammation and facilitate weaning from mechanical ventilation in infants with evolving BPD. Early clinical trials demonstrated that dexamethasone improves lung mechanics and increases the likelihood of successful extubation in ventilator-dependent infants (7–9). However, concerns regarding potential adverse neurodevelopmental outcomes led to a reduction in routine early steroid use and prompted the development of lower dose regimens and more selective treatment strategies (10, 11). Subsequent studies have evaluated both dexamethasone and hydrocortisone as therapeutic approaches aimed at reducing BPD severity while limiting cumulative steroid exposure (12).

Two corticosteroid regimens have been particularly influential in neonatal practice. The DART (Dexamethasone: A Randomized Trial) study introduced a low-dose dexamethasone regimen designed to facilitate extubation in infants with persistent ventilator dependence while minimising total steroid exposure (13). The PREMILOC (early low-dose hydrocortisone in extremely preterm infants) trial subsequently evaluated early low-dose hydrocortisone administered during the first week of life in extremely preterm infants at risk of BPD (14). Although these trials demonstrated respiratory benefits, translation of these regimens into routine clinical practice has been variable across neonatal units.

Observational studies from large neonatal cohorts have shown that corticosteroid administration frequently reflects underlying respiratory disease severity and evolving clinical trajectory, with treatment decisions guided by individual clinical assessment rather than standardized protocols (15–18). Consequently, real-world analyses are essential to understand how corticosteroids are used in neonatal care and how steroid exposure relates to respiratory outcomes in clinical practice. This is particularly important in observational datasets, where treatment allocation is not random, may reflect escalating respiratory severity, and requires careful interpretation of associations. Because corticosteroids are typically administered to infants with the most severe and persistent respiratory disease, observed associations between steroid exposure and outcomes are likely to reflect treatment selection within a high-risk population rather than causal treatment effects (19).

The aim of this study was to examine real-world patterns of postnatal corticosteroid exposure in preterm infants born before 32 weeks of gestation over a nine-year period. We also evaluated associations between steroid therapy and respiratory outcomes, including BPD severity, duration of respiratory support, home oxygen requirement, and mortality before 36 weeks’ postmenstrual age. Few studies have described corticosteroid prescribing patterns alongside detailed respiratory outcomes within a single-centre cohort over an extended period. This study is intended to characterize contemporary corticosteroid prescribing patterns and to inform interpretation of associated outcomes in observational datasets, rather than to evaluate treatment efficacy.

## Methods

### Study design and setting

This retrospective cohort was conducted in the neonatal intensive care unit at King Abdulaziz Medical City, Riyadh, Saudi Arabia, a tertiary referral centre providing advanced care for extremely preterm and critically ill neonates. Infants born before 32 completed weeks of gestation were included over a nine-year period from January 2017 to December 2025.

### Study population

All infants born at less than 32 completed weeks of gestation and admitted to the neonatal intensive care unit during the study period were screened for eligibility. Infants were identified from the institutional neonatal clinical database and cross-checked against electronic medical records.

Infants with major congenital anomalies, chromosomal or genetic disorders, or major congenital heart disease were excluded. Following application of eligibility criteria and data verification, the final analytic cohort consisted of 1,228 infants.

### Definition of postnatal steroid exposure

The primary exposure was systemic postnatal corticosteroid therapy administered for evolving or established BPD. Steroid exposure was categorized according to the pharmacological regimen received during the neonatal admission.

Four exposure groups were defined: no postnatal steroids, hydrocortisone only, dexamethasone only, and combined exposure to both hydrocortisone and dexamethasone during the same admission. For analyses evaluating overall steroid exposure, infants receiving any corticosteroid were grouped together and compared with those who did not receive steroid therapy.

In the study unit, hydrocortisone was typically initiated during the first week of life in extremely preterm infants considered at high risk of evolving BPD, following dosing principles derived from the PREMILOC regimen. This consisted of 1 mg/kg every 12 h for 7 days, followed by 0.5 mg/kg every 12 h for 3 days. Early hydrocortisone based on PREMILOC principles was introduced into clinical practice in our unit after publication of the trial in 2016, with increasing adoption over the study period (14). Steroid initiation was based on individual senior clinician decision-making rather than a formalised risk score–based protocol.

Dexamethasone was generally administered after the first week of life in infants with persistent ventilator dependence despite standard respiratory management, following the DART protocol. This consisted of a 10-day tapering course with a cumulative dose of approximately 0.89 mg/kg (0.15 mg/kg/day for 3 days, 0.10 mg/kg/day for 3 days, 0.05 mg/kg/day for 2 days, and 0.02 mg/kg/day for 2 days).

The no postnatal steroids group refers to infants who did not receive systemic corticosteroids for respiratory indications during their neonatal admission. Corticosteroid use for non-respiratory indications (e.g., hypotension) was outside the scope of this study and was not included in the exposure classification.

Treatment decisions were made by the attending clinical team based on routine clinical assessment.

### Clinical variables

Baseline maternal and neonatal characteristics were extracted from the institutional neonatal database. Maternal variables included mode of delivery, premature rupture of membranes, maternal diabetes, maternal hypertension, and clinical chorioamnionitis.

Neonatal variables included gestational age, birth weight, sex, and 5-minute Apgar score. Antenatal corticosteroid exposure was recorded as completion of a full course.

Respiratory variables included surfactant administration and initial respiratory support at admission, categorised as continuous positive airway pressure, non-invasive mechanical ventilation, conventional mechanical ventilation, or high-frequency oscillatory ventilation.

Study era was defined according to calendar periods to account for temporal changes in respiratory management and corticosteroid prescribing practices within the unit.

### Outcome measures

The primary outcomes were BPD severity at 36 weeks’ postmenstrual age, death before 36 weeks’ postmenstrual age, and the composite outcome of BPD or death. Additional outcomes included duration of invasive mechanical ventilation, duration of non-invasive respiratory support, and requirement for home oxygen therapy at discharge.

BPD severity was classified according to established grading criteria (Jensen classification) and analysed as an ordinal outcome (17). Duration of respiratory support was defined as the cumulative number of days of invasive mechanical ventilation and non-invasive respiratory support during the hospital admission and analysed as count outcomes.

Home oxygen requirement at discharge, death before 36 weeks’ postmenstrual age, and the composite outcome of BPD or death were analysed as binary outcomes.

### Statistical analysis

Continuous variables were summarized as medians with interquartile ranges due to non-normal distributions. Comparisons between groups were performed using the Mann–Whitney U test for continuous variables and the chi-square test for categorical variables.

Baseline characteristics were compared between infants who received postnatal corticosteroids and those who did not to assess differences in clinical characteristics and potential confounding by indication. Patterns of steroid use were also examined across gestational age strata, study era, and initial respiratory support categories.

Unadjusted comparisons of respiratory outcomes were performed across steroid exposure groups to describe crude differences in BPD severity, duration of respiratory support, home oxygen requirement, death before 36 weeks’ postmenstrual age, and the composite outcome of BPD or death. Multivariable regression models were then constructed to evaluate adjusted associations between steroid exposure and clinical outcomes.

BPD severity at 36 weeks’ postmenstrual age was analyzed using ordinal logistic regression. Binary logistic regression models were used to evaluate associations with home oxygen requirement at discharge, death before 36 weeks’ postmenstrual age, and the composite outcome of BPD or death. Duration of invasive and non-invasive respiratory support was analyzed using negative binomial regression models because of overdispersion of count data.

All multivariable models were adjusted for clinically relevant covariates selected *a priori*, including gestational age, sex, complete antenatal corticosteroid exposure, premature rupture of membranes, surfactant administration, study era, and initial respiratory support category. Effect estimates from logistic regression models are reported as adjusted odds ratios with 95% confidence intervals, and estimates from negative binomial models are reported as incidence rate ratios with 95% confidence intervals.

Given the non-random allocation of postnatal corticosteroid therapy, confounding by indication, whereby infants with greater illness severity are more likely to receive corticosteroid therapy, was addressed using complementary approaches. Baseline characteristics were compared between exposure groups to characterise differences in clinical severity. In addition, propensity score weighting was performed as a sensitivity analysis. Propensity scores estimating the probability of receiving corticosteroid therapy were derived from baseline variables associated with respiratory disease severity, including gestational age, birth weight, sex, antenatal corticosteroid exposure, and surfactant administration. Inverse probability weighting based on these scores was applied in regression models evaluating the composite outcome.

Sensitivity analyses were performed according to steroid formulation, with separate models comparing hydrocortisone, dexamethasone, and combined exposure with no steroid exposure for BPD severity, the composite outcome of BPD or death, and duration of invasive ventilation.

All analyses were performed using IBM SPSS Statistics version 31 (IBM Corp, Armonk, New York, USA). Two-sided *p* values <0.05 were considered statistically significant.

### Data management and missing data

Clinical data were extracted from the institutional neonatal database and verified against electronic medical records where required. Data quality checks included range validation, internal consistency assessment, and verification of key exposure and outcome variables.

Complete case analysis was used for regression models. Continuous variables with missing values were summarised using available case analysis. Propensity score–weighted analyses were performed on the largest available dataset.

## Results

A total of 1,228 preterm infants born at <32 weeks’ gestation were included in the analysis, of whom 326 (26.5%) received postnatal corticosteroids. Hydrocortisone was administered in 9.5% of infants, dexamethasone in 15.2%, and both in 1.8%. Baseline characteristics differed between exposure groups, reflecting differences in gestational age, birth weight, and respiratory disease severity. Patterns of steroid use varied across gestational age strata, study era, and initial respiratory support. Unadjusted and adjusted analyses of respiratory outcomes are presented below.

Table 1 shows baseline characteristics according to postnatal steroid exposure. Infants who received postnatal steroids were more premature and had lower birth weight compared with those without steroid exposure (median gestational age 26 vs. 29 weeks; birth weight 800 vs. 1,210 g; both *p* < 0.001). They also had lower 5-minute Apgar scores (*p* < 0.001). Steroid-exposed infants were more likely to have received surfactant therapy (89.9% vs. 60.5%, *p* < 0.001) and invasive respiratory support at admission, including conventional ventilation (68.1% vs. 35.8%) and HFOV (13.8% vs. 6.5%) (all *p* < 0.001), while non-invasive support was less frequent. Length of stay was longer among infants receiving steroids (94.5 vs. 42 days, *p* < 0.001). PROM and cesarean delivery differed between groups (*p* = 0.012 and *p* = 0.034, respectively), whereas sex and maternal comorbidities were similar.

**Table 1:** Baseline characteristics according to any postnatal steroid exposure.

Characteristic	No postnatal steroids (*n* = 902)	Any postnatal steroids (*n* = 326)	*p* value
Gestational age, weeks	29 [28, 30]	26 [24, 28]	<0.001
Birth weight, g	1,210 [990, 1,500]	800 [650, 1,000]	<0.001
5-min Apgar score	8 [7, 9]	7 [6, 8]	<0.001
Male sex, *n* (%)	497 (55.1%)	179 (54.9%)	1.000
Cesarean delivery, *n* (%)	611 (67.7%)	199 (61.0%)	0.034
PROM, *n* (%)	265 (29.4%)	121 (37.1%)	0.012
Maternal diabetes, *n* (%)	155 (17.2%)	50 (15.3%)	0.497
Maternal hypertension, *n* (%)	127 (14.1%)	41 (12.6%)	0.560
Chorioamnionitis, *n* (%)	47 (5.2%)	27 (8.3%)	0.063
Complete antenatal steroids, *n* (%)	546 (60.5%)	214 (65.6%)	0.118
Any surfactant administered, *n* (%)	546 (60.5%)	293 (89.9%)	<0.001
Length of stay, days	42 [29, 61]	94.5 [63.2, 118.8]	<0.001
Initial respiratory support: CPAP, *n* (%)	205 (22.7%)	25 (7.7%)	<0.001
Initial respiratory support: NIMV, *n* (%)	315 (34.9%)	34 (10.4%)	<0.001
Initial respiratory support: Conventional, *n* (%)	323 (35.8%)	222 (68.1%)	<0.001
Initial respiratory support: HFOV, *n* (%)	59 (6.5%)	45 (13.8%)	<0.001

Data are presented as median [interquartile range] or *n* (%). PROM, premature rupture of membranes; CPAP, continuous positive airway pressure; NIMV, non-invasive mechanical ventilation; HFOV, high-frequency oscillatory ventilation.

Table 2 shows patterns of postnatal steroid use across gestational age, study era, and initial respiratory support. Steroid exposure varied across gestational age strata (*p* < 0.001), with higher use among infants born before 26 weeks, where 54.4% received steroids compared with 7.0% among those 30–31 weeks. Steroid prescribing differed across study eras (*p* < 0.001), with dexamethasone use increasing from 7.3% in 2017–2019 to 20.1% in 2023–2025. Steroid exposure was more frequent among infants receiving invasive respiratory support (*p* < 0.001), particularly conventional ventilation (40.7% received any steroids) and HFOV (43.3%), compared with those managed with CPAP (10.9%) or NIMV (9.7%).

**Table 2:** Practice patterns of postnatal steroid use by gestational age, study era, and initial respiratory support.

Stratum	Total n	None	Hydrocortisone	Dexamethasone	Both
Overall cohort	1,228	902 (73.5%)	117 (9.5%)	187 (15.2%)	22 (1.8%)
Gestational age <26	239	109 (45.6%)	37 (15.5%)	80 (33.5%)	13 (5.4%)
Gestational age 26–27	219	112 (51.1%)	53 (24.2%)	48 (21.9%)	6 (2.7%)
Gestational age 28–29	313	256 (81.8%)	21 (6.7%)	35 (11.2%)	1 (0.3%)
Gestational age 30–31	457	425 (93.0%)	6 (1.3%)	24 (5.3%)	2 (0.4%)
Study era 2017–2019	518	431 (83.2%)	41 (7.9%)	38 (7.3%)	8 (1.5%)
Study era 2020–2022	312	218 (69.9%)	22 (7.1%)	69 (22.1%)	3 (1.0%)
Study era 2023–2025	398	253 (63.6%)	54 (13.6%)	80 (20.1%)	11 (2.8%)
Initial support CPAP	230	205 (89.1%)	4 (1.7%)	21 (9.1%)	0 (0%)
Initial support NIMV	349	315 (90.3%)	13 (3.7%)	20 (5.7%)	1 (0.3%)
Initial support Conventional	545	323 (59.3%)	84 (15.4%)	119 (21.8%)	19 (3.5%)
Initial support HFOV	104	59 (56.7%)	16 (15.4%)	27 (26.0%)	2 (1.9%)

Data are presented as *n* (% within row). CPAP, continuous positive airway pressure; NIMV, non-invasive mechanical ventilation; HFOV, high-frequency oscillatory ventilation. *p* values derived from chi-square tests.

Table 3 shows unadjusted respiratory outcomes according to postnatal steroid formulation. BPD severity differed across groups (*p* < 0.001), with higher proportions of grade 2–3 BPD among infants receiving dexamethasone (43.7%) or both steroids (68.2%) compared with those receiving no steroids (8.3%). Median duration of invasive ventilation increased across groups from 4 days in infants without steroid exposure to 13 days with hydrocortisone, 30 days with dexamethasone, and 47 days in those receiving both steroids (*p* < 0.001). Non-invasive respiratory support days also differed (*p* < 0.001), with higher values in steroid-treated groups. The proportion of infants requiring home oxygen was higher with dexamethasone (4.4%) compared with no steroids (0.4%) (*p* < 0.001). The composite outcome of BPD or death increased from 28.0% in the no-steroid group to 68.4% with hydrocortisone, 65.2% with dexamethasone, and 86.4% with combined exposure (*p* < 0.001), while death before 36 weeks differed between groups (*p* = 0.014).

**Table 3:** Unadjusted respiratory outcomes according to postnatal steroid formulation.

Steroid group	n	BPD grade distribution	Total intubation days	Total non-invasive days	Home oxygen	Death <36 weeks	BPD/death composite
None	902	G0 655 (81.4%); G1 83 (10.3%); G2 61 (7.6%); G3 6 (0.7%)	4 [1, 9]	4 [2, 11]	3 (0.4%)	96 (10.7%)	253 (28.0%)
Hydrocortisone	117	G0 35 (36.8%); G1 23 (24.2%); G2 33 (34.7%); G3 4 (4.2%)	13 [5, 34]	20 [8, 34]	3 (2.8%)	20 (17.2%)	80 (68.4%)
Dexamethasone	187	G0 65 (37.4%); G1 33 (19.0%); G2 61 (35.1%); G3 15 (8.6%)	30 [9, 44]	16 [7, 31.8]	8 (4.4%)	13 (7.0%)	122 (65.2%)
Both	22	G0 3 (13.6%); G1 4 (18.2%); G2 11 (50.0%); G3 4 (18.2%)	47 [33.2, 68]	23 [14, 40]	0 (0%)	0 (0%)	19 (86.4%)

Data are presented as median [interquartile range] or *n* (%). BPD, bronchopulmonary dysplasia.

Table 4 shows adjusted associations between postnatal steroid exposure and clinical outcomes. Steroid exposure was associated with higher BPD severity at 36 weeks (OR 2.71, 95% CI 1.92–3.81, *p* < 0.001) and increased likelihood of home oxygen at discharge (OR 6.36, 95% CI 1.47–27.64, *p* = 0.014). The composite outcome of BPD or death was also higher among steroid-exposed infants (OR 1.98, 95% CI 1.41–2.78, *p* < 0.001). In contrast, steroid exposure was associated with lower odds of death before 36 weeks’ postmenstrual age (OR 0.25, 95% CI 0.15–0.41, *p* < 0.001).

**Table 4:** Adjusted associations between postnatal steroid exposure and clinical outcomes.

Outcome	Model	Adjusted OR	95% CI	*p* value	N analysed
Higher BPD grade at 36 weeks	Ordinal logistic regression	2.71	1.92 to 3.81	<0.001	1,096
Home oxygen at discharge	Binary logistic regression	6.36	1.47 to 27.64	0.014	1,151
Death before 36 weeks PMA	Binary logistic regression	0.25	0.15 to 0.41	<0.001	1,220
BPD or death before 36 weeks	Binary logistic regression	1.98	1.41 to 2.78	<0.001	1,228

Models adjusted for gestational age, sex, complete antenatal steroid exposure, PROM, surfactant exposure, study era, and initial respiratory support category. OR, odds ratio; CI, confidence interval; BPD, bronchopulmonary dysplasia.

Table 5 shows adjusted associations between postnatal steroid exposure and duration of respiratory support. Steroid exposure was associated with longer duration of invasive ventilation (IRR 2.65, 95% CI 2.25–3.11, *p* < 0.001). No significant association was observed between steroid exposure and duration of non-invasive respiratory support (IRR 1.01, 95% CI 0.85–1.21, *p* = 0.874).

**Table 5:** Adjusted associations between postnatal steroid exposure and duration of respiratory support.

Outcome	Model	Effect estimate (IRR)	95% CI	*p* value	N analysed
Total days intubated	Negative binomial regression	2.65	2.25 to 3.11	<0.001	896
Total non-invasive support days	Negative binomial regression	1.01	0.85 to 1.21	0.874	1,092

Models adjusted for gestational age, sex, complete antenatal steroid exposure, PROM, surfactant exposure, study era, and initial respiratory support category. IRR, incidence rate ratio; CI, confidence interval.

Table 6 shows formulation-specific sensitivity analyses comparing hydrocortisone, dexamethasone, and combined exposure with no postnatal steroids. Both hydrocortisone (OR 2.37, 95% CI 1.51–3.73, *p* < 0.001) and dexamethasone (OR 2.75, 95% CI 1.86–4.06, *p* < 0.001) were associated with higher BPD severity, while combined exposure showed higher effect estimates (OR 5.67, 95% CI 2.40–13.39, *p* < 0.001). Similar patterns were observed for the composite outcome of BPD or death, with higher estimates for combined exposure (OR 4.03, 95% CI 1.01–16.05, *p* = 0.048). Duration of invasive ventilation was also increased across all formulations, with the largest effect observed in the combined group (IRR 4.35, 95% CI 2.79–6.80, *p* < 0.001).

**Table 6:** Formulation-specific sensitivity analyses of postnatal steroid exposure.

Outcome	Comparison	Adjusted OR/IRR	95% CI	*p* value
Higher BPD grade	Hydrocortisone vs. none	2.37	1.51 to 3.73	<0.001
Higher BPD grade	Dexamethasone vs. none	2.75	1.86 to 4.06	<0.001
Higher BPD grade	Both vs. none	5.67	2.40 to 13.39	<0.001
BPD or death before 36 weeks	Hydrocortisone vs. none	1.90	1.18 to 3.07	0.008
BPD or death before 36 weeks	Dexamethasone vs. none	1.94	1.29 to 2.92	0.002
BPD or death before 36 weeks	Both vs. none	4.03	1.01 to 16.05	0.048
Total days intubated	Hydrocortisone vs. none	2.06	1.66 to 2.56	<0.001
Total days intubated	Dexamethasone vs. none	2.84	2.35 to 3.44	<0.001
Total days intubated	Both vs. none	4.35	2.79 to 6.80	<0.001

Models adjusted for gestational age, sex, complete antenatal steroid exposure, PROM, surfactant exposure, study era, and initial respiratory support category. BPD, bronchopulmonary dysplasia; CI, confidence interval. Higher BPD grade’ represents increasing severity across the ordinal BPD classification (Jensen criteria).

Table 7 shows the propensity-weighted sensitivity analysis for the composite outcome of BPD or death before 36 weeks. After inverse probability weighting, postnatal steroid exposure remained associated with the composite outcome (OR 1.90, 95% CI 1.53–2.34, *p* < 0.001).

**Table 7:** Propensity-weighted sensitivity analysis of postnatal steroid exposure and composite outcome.

Outcome	Adjusted OR	95% CI	*p* value
BPD or death before 36 weeks	1.90	1.53 to 2.34	<0.001

Propensity scores were derived from baseline variables including gestational age, birth weight, sex, antenatal steroid exposure, and surfactant administration. CI, confidence interval.

## Discussion

This study examined real-world patterns of postnatal corticosteroid therapy and associated respiratory outcomes in a large cohort of preterm infants born before 32 weeks’ gestation over a nine-year period in a tertiary neonatal intensive care unit. Three principal findings were observed. First, postnatal steroid exposure was concentrated among infants of lower gestational age and among those requiring invasive respiratory support. Second, infants who received postnatal steroids had higher BPD severity, longer duration of invasive ventilation, and were more likely to require home oxygen therapy at discharge, consistent with higher observed respiratory morbidity rates within this higher-risk population. Third, these associations remained evident after multivariable adjustment and propensity-weighted analyses designed to address confounding by indication. Overall, the findings suggest that systemic corticosteroid therapy in this cohort was preferentially administered to infants with more severe and persistent respiratory disease, reflecting treatment selection within a higher-risk clinical population rather than random treatment allocation.

The findings of this cohort should be interpreted in the context of the pathophysiology of BPD and the clinical role of postnatal corticosteroids. Bronchopulmonary dysplasia reflects disrupted lung development driven by the combined effects of prematurity, oxygen exposure, mechanical ventilation, and inflammation (1, 2, 20). Because inflammation contributes to ongoing lung injury, systemic corticosteroids have been used to facilitate weaning from mechanical ventilation in infants with evolving disease (7, 9). In the present study, infants who received postnatal steroids had lower gestational age, lower birth weight, and higher requirements for invasive respiratory support at baseline, together with greater subsequent respiratory morbidity. This pattern is consistent with prior observational and network studies demonstrating that corticosteroid therapy is typically administered to infants with persistent ventilator dependence or more severe respiratory disease (12, 15, 17). These findings highlight the well-recognised challenge of confounding by indication in observational studies of steroid therapy, whereby infants receiving corticosteroids represent a subgroup with greater baseline respiratory risk. Accordingly, observed differences in outcomes likely reflect underlying illness severity and treatment selection rather than direct treatment effects.

The present analysis also provides insight into how corticosteroid regimens are applied in contemporary neonatal care in a tertiary centre. In this cohort, hydrocortisone therapy broadly reflected dosing strategies derived from the PREMILOC trial, which evaluated early low-dose hydrocortisone administered during the first week of life in extremely preterm infants at risk of BPD (14). In contrast, dexamethasone therapy followed principles similar to the DART protocol, a low-dose regimen designed to facilitate extubation in infants with persistent ventilator dependence beyond the first week of life (13). These approaches reflect a shift towards lower cumulative steroid exposure and more selective use in infants with evolving or established respiratory disease.

This change in practice has been informed by evidence from randomized trials and systematic reviews indicating that later, lower-dose dexamethasone regimens may support extubation while reducing the risk of adverse neurodevelopmental outcomes compared with earlier high-dose strategies (9, 13). In addition, hydrocortisone has been increasingly evaluated as an alternative corticosteroid with potentially different neurodevelopmental and cardiovascular safety profiles, particularly in early evolving disease (14, 21). Nevertheless, translation of trial-based protocols into clinical practice remains heterogeneous across neonatal centres. Observational studies and international surveys have demonstrated substantial variation in timing, dosing, and choice of corticosteroid, reflecting differences in local protocols, clinician preferences, and perceived risk–benefit balance (12, 22–24). Furthermore, meta-analyses indicate that the effectiveness and safety of postnatal steroid therapy are strongly influenced by timing of initiation, cumulative dose, and baseline risk of BPD, supporting an individualized approach to treatment rather than uniform application (24, 25). These considerations provide important context for interpreting the findings of the present study, in which steroid exposure reflects real-world clinical decision-making within a heterogeneous population rather than standardized protocol-driven treatment.

The association between postnatal steroid exposure and prolonged invasive ventilation observed in this cohort is likely to reflect the clinical context in which corticosteroids are initiated. In many neonatal intensive care settings, systemic steroids are administered to infants who remain ventilator dependent despite standard respiratory management. Randomized trials have demonstrated that dexamethasone therapy can facilitate extubation in selected infants with evolving BPD (7, 13), but these studies enrolled infants who were already ventilator dependent and therefore at high risk of prolonged respiratory support. Observational analyses have similarly shown that corticosteroid therapy is frequently used as rescue treatment in infants with persistent respiratory failure (15, 26).

Within this context, the longer duration of invasive ventilation observed among steroid-treated infants likely reflects underlying disease severity and treatment timing rather than a direct effect of therapy. A similar consideration applies to the lower adjusted odds of death before 36 weeks’ postmenstrual age observed in this study. The observed lower mortality among steroid-exposed infants should be interpreted cautiously and likely reflects treatment selection and survivor bias, as corticosteroids are typically administered to infants who survive the early phases of severe illness and remain candidates for ongoing intensive care. Comparable patterns have been reported in neonatal cohort studies, where differences in mortality largely reflect treatment eligibility and timing rather than causal effects of corticosteroids (12, 27).

Gestational age stratified analyses further illustrate the strong maturity gradient in postnatal steroid use. In this cohort, steroid treatment occurred predominantly among infants born before 28 weeks’ gestation and was uncommon among more mature infants. This pattern is consistent with previous reports demonstrating that both the incidence of BPD and the likelihood of receiving systemic corticosteroids increase with decreasing gestational age (16, 28–30). These observations should be interpreted within the broader evidence base showing that respiratory outcomes in preterm infants are strongly influenced by gestational maturity, with lower gestational age associated with greater risk of BPD, prolonged ventilator dependence, and mortality.

The results of this study should be considered in the context of the existing literature on postnatal corticosteroid therapy in preterm infants. While randomized trials and network analyses have primarily focused on the efficacy and safety of corticosteroids for evolving BPD, fewer studies have described real-world patterns of steroid prescribing across gestational age groups and respiratory support strategies. This cohort extends existing evidence by characterising contemporary steroid utilization alongside detailed respiratory outcomes in a large population of preterm infants, demonstrating selective use in higher-risk infants rather than uniform application across the population.

This study has several strengths. It includes a large cohort of preterm infants managed within a single tertiary neonatal intensive care unit over a nine-year period, allowing evaluation of contemporary practice patterns across different gestational age groups and respiratory support strategies. The dataset enabled detailed characterization of postnatal corticosteroid exposure, including formulation and associated respiratory outcomes. The analytical approach incorporated multivariable regression models and propensity score weighting to address confounding by indication, which is a major limitation in observational studies of steroid therapy. These methods improve the robustness of the findings by accounting for key baseline differences between treated and untreated infants, although they do not eliminate bias related to unmeasured factors.

Several limitations should be considered. The retrospective design limits the ability to capture all clinical factors that influence the decision to initiate corticosteroid therapy, including detailed measures of illness severity, ventilator settings, and clinician judgement at the bedside. Although we adjusted for key markers of illness severity, residual confounding and selection effects inherent to observational data remain possible**.** The timing of steroid initiation relative to the course of illness could not be evaluated and may introduce bias, as infants must survive long enough and remain clinically stable to receive treatment. Neurodevelopmental follow-up data were not available, and therefore the study cannot address long-term safety outcomes associated with postnatal corticosteroid treatment. In addition, the study reflects the experience of a single tertiary centre with specific clinical protocols, which may limit generalizability to other settings, particularly in light of known intercentre variation in BPD incidence and management practices (31). In addition, the study period overlapped with the COVID-19 pandemic, a time associated with broader system-level pressures and potential changes in clinical practice, which may have influenced patterns of care despite adjustment for study era in the analysis.

## Conclusions

In this large single-centre cohort of preterm infants born before 32 weeks’ gestation, postnatal corticosteroid therapy was used primarily in infants with lower gestational age and greater respiratory support requirements. Infants who received steroids had higher bronchopulmonary dysplasia severity, longer duration of invasive ventilation, and increased likelihood of home oxygen therapy at discharge, while lower mortality was observed among steroid-exposed infants. These patterns persisted after multivariable adjustment and propensity-weighted analyses and are consistent with treatment selection in a higher-risk population rather than uniform treatment effects. Overall, these findings reflect real-world corticosteroid use in higher-risk infants and should be interpreted as associations within a selected population rather than causal treatment effects.

## Data Availability

The raw data supporting the conclusions of this article will be made available by the authors, without undue reservation.
